# Copper stress induces antioxidant responses and accumulation of sugars and phytochelatins in Antarctic *Colobanthus quitensis* (Kunth) Bartl.

**DOI:** 10.1186/s40659-018-0197-0

**Published:** 2018-11-14

**Authors:** Rodrigo A. Contreras, Marisol Pizarro, Hans Köhler, Claudio A. Sáez, Gustavo E. Zúñiga

**Affiliations:** 1 Laboratorio de Fisiología y Biotecnología Vegetal, Departamento de Biología, Facultad de Química y Biología, Universidad de Santiago de Chile, L.B. O’Higgins #3363, Estación Central, Santiago, Chile; 2 Centro para el Desarrollo de la Nanociencia y la Nanotecnología (CEDENNA), Universidad de Santiago de Chile, Santiago, Chile; 3 Laboratory of Aquatic Environmental Research (LACER), Center of Advanced Studies, Universidad de Playa Ancha, Viña del Mar, Chile

**Keywords:** *C. quitensis*, Metal, Antioxidants, Phytochelatins, Sugars, Antarctica

## Abstract

**Background:**

In field, *C. quitensis* is subjected to many abiotic extreme environmental conditions, such as low temperatures, high UV-B, salinity and reduced water potentials, but not metal or metalloid high concentrations in soil, however, other members of Caryophyllaceae family have tolerance to high concentrations of metals, this is the case of *Silene* genre. In this work, we hypothesize that *C. quitensis* have the same mechanisms of *Silene* to tolerate metals, involving accumulation and induction of antioxidant systems, sugar accumulation and the induction of thiols such as phytochelatins to tolerate.

**Results:**

The results showing an effective antioxidant defensive machinery involving non-enzymatic antioxidants such as phenolics, GSH and ascorbic acid, in another hand, GSH-related oligomers (phytochelatins) and sugars was induced as a defensive mechanism.

**Conclusions:**

*Colobanthus quitensis* exhibits certain mechanisms to tolerate copper in vitro demonstrating its plasticity to tolerate several abiotic stress conditions.

## Introduction

*Colobanthus quitensis* (Kunth) Bartl. (Caryophyllaceae) is one of the two vascular plants that inhabit naturally the Maritime Antarctica [1]. Antarctica is an extreme environment that exhibits many extreme conditions that have direct influence in plant physiology, growth, reproduction and survival, the principal conditions are low temperatures, high UV-B radiation, salinity, low water status in soils and very low PAR in winter [2]. Additionally, in King George Island (Maritime Antarctic), soils show concentrations of cupric ion around of 2.0 ± 0.27 mmol kg^−1^ [3]. The tolerance to all these conditions is natural in this specie and converts it in an interesting model to study mechanisms of tolerance to abiotic stress.

The presence of toxic compounds in the soil, such as heavy metals, is one important factor that can cause damage to plants by altering major plant physiological and metabolic processes [4]. Depending on their oxidation states, heavy metals can be highly reactive, resulting in toxicity of plant cells in many ways. At the cellular and molecular level, toxicity results in alterations of different plant physiological processes, including inactivation and denaturation of enzymes, proteins, blocking of functional groups of metabolically important molecules, displacement/substitution of essential metal ions from biomolecules and functional cellular units, conformational modifications and disruption of membrane integrity [4], which is finally attributed to altered plant metabolism, inhibition of photosynthesis, respiration, and the activities of several key enzymes [5]. In addition, heavy metals are known to disturb redox homeostasis by stimulating the formation of free radicals and ROS such as $\text{O}_{2}^{ \cdot - }$, ^1^O_2_, H_2_O_2_, and OH· [5, 6]. This increase in ROS exposes cells to oxidative stress leading to lipid peroxidation, biological macromolecule deterioration, membrane dismantling, ion leakage, and DNA-strand cleavage and finally death of plants [7].

Plants employ various strategies to cope with the toxic effects of metals. Resistance to heavy metals stress can be achieved by “tolerance” when plants survive in the presence of high internal metal concentration. In this situation metals are intracellularly chelated through the synthesis of amino acids, organic acids, GSH, or heavy metal-binding ligands such as MTs, the YSL proteins that moves systemically the copper using the ubiquitous chelator NA, the PCs, compartmentation within vacuoles, and upregulation of the antioxidant defense and glyoxalase systems to counter the deleterious effects caused by ROS [8–10].

Plant-metal interaction have similar mechanisms of another plant-abiotic conditions, and responses involves a defensive enzymatic and non-enzymatic antioxidant systems [11], involving for example, the Asc-GSH cycle, to detoxifying of damaging levels of ROS generated by electronic decoupling in chloroplast and mitochondria, and for another font such as Fenton reaction [12]. In the Asc-GSH cycle a few enzymes control partially the levels of ROS and maintains them in harmless levels, which allows the cell still alive in spite an adverse condition. The enzymatic antioxidant machinery system comprises enzymes from Asc-GSH cycle, and other enzymes that act independent from this cycle such as SOD, CAT, and POD. Superoxide anions generated are converted to H_2_O_2_ by the action of SOD, and in the meanwhile the increasing of H_2_O_2_ is avoided by the activities of APX, CAT, POD and GPX. Finally, the balance between ROS generation and control determines the chance to survival of the organism subjected to stress [13]. Another known way to prevent the excess of ROS is the non-enzymatic mechanism that involves Asc, GSH and other metabolites becoming from secondary metabolism, principally from phenylpropanoid pathway. These metabolites act as scavengers of ROS, and in conjunct to the conjunct of antioxidant enzymes provides a powerful hardware to attenuate ROS, and both are crucial to mediates the survival [14].

Copper is an essential micronutrient used in several electron transport reactions including the catalysis of redox reactions in mitochondria and chloroplasts [15]. However, at high levels copper turns toxic inducing the increasing of ROS levels within subcellular compartments [16]. It is known that the Mehler reaction is inhibited by high levels of copper on the PSI and it seems that copper has a negative effect in the Hill reaction on PSII too, leading to changes in the carbon metabolism [17] affecting not only directly cellular mechanisms of response, but also in an indirect manner, the signal transduction into the plant cell [18], that drives to changes in carbohydrate metabolism, because the influence of the increasing ROS levels [19].

Plants also have responses that involve direct mechanisms to survive to heavy metal toxicity. One of the most important mechanism of heavy-metal detoxification is the chelation of metals through ligands derived of GSH [8], where PCs stand as an significant inducible group of heavy-metal-binding ligands, that belong to a family of non-peptidic bonded peptides that consist in repetitions of (γ-Glu-Cys)n-Gly (n = 2–11). PCs are synthetized from GSH by the PC synthase, a constitutive enzyme that requires post-translational modification to perform as an active enzyme [20].

The relevance of changes in both plant metabolisms, primary and secondary, can drive to responses that allow the survivor of plants exposed to heavy-metal conditions. Based in the mechanisms described, we postulate that *C. quitensis*, has mechanisms to tolerate copper stress, because it has the machinery to tolerate a wide range of different abiotic conditions.

## Materials and methods

### Plant material

In vitro shoots were generated about previously described [21]. The explants were growth during 1 month in a Murashige-Skoog [22] basal media, supplemented with phytohormones *N*^*6*^-benzilaminopurine (0.3 mg L^−1^) and kinetin (0.1 mg L^−1^), using 0.2% of phytagel (Sigma-Aldrich, St. Louis, MO, USA) as gelling agent at pH 4.5 ± 2, in a conservation chambers at 13 ± 2 °C, with a photoperiod of 16/8 h light/darkness. Then the month, the explants was transferred to a culture media supplemented with 150 and 300 µM of copper (II) sulfate, and the explants was exposed for 15 days.

### Oxidative damage parameters

The total ROS was measured using the spectrofluorometric method [23], 100 mg of fresh explants was incubated 1 h in 1 mL of 10 µM DCHF-DA solution in Tris–HCl (50 mM, pH 8.0), then the tissue was washed with EDTA 10 mM and ground with liquid nitrogen to fine powder. The fine powder was resuspended in 1 mL of Tris–HCl (50 mM, pH 8.0) and was filtered in Wathman no. 2 paper, finally was measure the fluorescence intensity (LS-5, Perkin-Elmer, Well., MA, USA) using 488 nm of exiting wavelength and 525 nm of emission wavelength. The results were expressed in equivalents of DCF. The TBARS was measured spectrophotometrically [24] for indicate membrane damage; 100 mg of fresh tissue was ground with liquid nitrogen to forming a fine powder, the powder was resuspended in 2 mL of 1% of TCA solution. The mixture was centrifuged at 10,000*g* for 5 min; 250 µL of supernatant was mixed with 1 mL of 0.5% of TBA in 20% of TCA solution. This mixture was incubated at 100 °C in a water bath for 30 min. Finally, was recording the absorbance at 532 and 600 nm, the results were expressed in MDA equivalents using the difference of A_532_–A_600_ with the molecular extinction coefficient 155 mM^−1^ cm^−1^ for the adduct formed by the TBA and MDA.

### Photosynthetic pigments content

The Chl-*a*, Chl-*b* and total carotenoids was measure using the spectrophotometrically method [25] the pigments were extracted using pure acetone, the mixture was sonicated (50-60 Hz) during 2 h at room temperature, the extract was diluted 10 times and was register the absorbance at 470, 649 and 665 nm. For the calculus was used the following equations:

1 $${\text{Chl}} - a\left( {\upmu{\text{g mL}}^{ - 1} } \right) = 1 3. 9 6\left( {{\text{A}}_{ 6 6 5} } \right) - 6. 8 8\left( {{\text{A}}_{ 6 4 9} } \right)$$

2 $${\text{Chl}} - b\left( {\upmu{\text{g mL}}^{ - 1} } \right) = 2 4. 9 6\left( {{\text{A}}_{ 6 4 9} } \right) - 7. 3 2\left( {{\text{A}}_{ 6 6 5} } \right)$$

3 $${\text{Total carotenoids }}\left( {\upmu{\text{g mL}}^{ - 1} } \right) = \left( { 100\left( {{\text{A}}_{ 4 70} } \right) - 2.0 5\left( {{\text{Chl}} - a} \right) - 1 1 4. 8\left( {{\text{Chl}} - b} \right)} \right)/ 2 4 5$$

### Protein extraction and antioxidant enzymes measurements

Proteins was extracted using 100 mg of fresh tissue ground in liquid nitrogen to fine powder and it was resuspended in 50 mM of Tris–HCl buffer (pH 7.5), the mixture was centrifuged at 4 °C 10 min at 10,000*g*, the supernatants correspond at soluble proteins. These proteins were quantified using modified Bradford method (900 µL of Bradford reagent, 80 µL of NaCl (aq) 150 mM and 20 µL of supernatants) after 2 min the absorbance at 595 nm was registered [26]. The concentration was calculated using BSA as standard. SOD (EC 1.15.1.1) activity was determined measuring the photochemical reduction NBT. The reaction mixture contains 600 µL of Tris–HCl (50 mM; pH 7) buffer, 10 µL of EDTA 10 mM, 100 µL of 130 mM methionine, 10 µL of 2 mM riboflavin and 200 µL of 3 mM of NBT and 100 µL of protein extract, the reaction mixture was incubated during 15 min at room temperature in light, blank corresponds a reaction mixture in darkness, we measure the absorbance of reduced NBT at 560 nm [27]. CAT (EC 1.11.1.6) activity was tested measuring using the decomposition of H_2_O_2_ at 240 nm for 60 s. The reaction mixture contains 1 mL of extraction buffer, 3 μL of H_2_O_2_ 30% and 20 μL of the supernatant [28]. Enzyme activity was calculated using a molar extinction coefficient of 39.4 mM^−1^ cm^−1^. The APX (EC 1.11.1.11) activity was tested measuring the decomposition of ascorbate at 290 nm for 60 s. The reaction mixture contained 1 mL of extraction buffer, 5 μL of H_2_O_2_ 30%, 40 μL of ascorbic acid 10 mM and 20 µL of the supernatant. Enzyme activity was calculated using a molar extinction coefficient of 2.8 mM^−1^ cm^−1^ [29]. The GR (EC 1.6.4.2) activity was determined by measuring the oxidation of NADPH at 340 nm for 3 min in 1 mL. The reaction mixture containing 1 mL of extraction buffer, 2 mM EDTA, NADPH 0.15 mM, 0.5 mM GSSG and 100 μL extract. Enzyme activity was calculated using a molar extinction coefficient of 6.2 mM^−1^ cm^−1^ [30]. Finally, the POD; (EC 1.11.1.7) was tested measuring the generation of tetraguaiacol at 470 nm for 60 s. The reaction mixture contains 1 mL of extraction buffer, 5 μL of H_2_O_2_ 30%, 5 μL of guaiacol and 10 μL of the supernatant. Enzyme activity was calculated using a molar extinction coefficient of 26.6 mM^−1^ cm^−1^ [28].

### Antioxidants extraction, non-enzymatic antioxidant parameters and total phenolic content

The non-enzymatic antioxidants were extracted using the hydroalcoholic solution (85% v/v of aqueous ethanol) with sonication per 2 h (50–60 Hz) at room temperature. First was measure the spectrophotometrically scavenge of DPPH· to form of DPPH_2_ at 517 nm for 4 min, the results was expressed in percentage of scavenge radical [31]. The total redactor power was measure using the FRAP assay, measuring spectrophotometrically reduction of Fe(III) to Fe(II) at 593 nm, using the capacity to form a blue complex with TPTZ for 4 min [32]. Finally, the total phenolics was measure using the phosphotungstomolybdic method, using the redox reaction with Folin-Ciocalteu’s reagent, using 100 µL of Folin-Ciocalteu’s reagent, with 500 µL of water and 100 µL of each extract for 15 min, the reaction was stopped with 300 µL of 7% of sodium carbonate solution, and was measure the total phenolics at 660 nm using gallic acid solution as standard [33].

### Total soluble sugars

We used the sulphuric anthrone method; we prepare a reaction mixture of 3 mL of sulphuric anthrone (1.5% of anthrone in concentred sulphuric acid) and 100 µL of hydroalcoholic extract, the reaction mixture was incubated at room temperature for 15 min and then it was register the absorbance at 620 nm. We used fructose as standard [2].

### Sugar determination

Sugars was analyzed using HPLC coupled to RID, aliquots of 100 µL of hydroethanolic extracts was lyophilized using a speed vac (Savant, Minn., USA), the pellet of sugars was resuspended in 100 µL of EDTA-Ca^2+^ (aq) (0.1 mM). 20 µL of samples was injected and separated using a Sugar-Pack column (6.5 × 300 mm) (Waters Corp., Massachusetts, USA) at 75 °C, the RID temperature was 55 °C and the mobile phase were an isocratic elusion solution of EDTA-Ca^2+^ (0.1 mM) with flow rate of 0.35 mL min^−1^ for 30 min. Pure standards of ascorbate, glucose, fructose, galactose, galactinol, sucrose, raffinose, stachyose, verbascose, xylose and lyxose were standardized and calibrated.

### GSH and phytochelatin determination

The analysis of phytochelatins was performed by the method previously described [34]. 100 mg of tissue was gowned in liquid nitrogen to form a fine powder and resuspended in 600 µL of 0.1% (w/v) of TFA with 6.3 mM of DTPA. The homogenate was transferred to test tube and centrifuged at 4 °C during 20 min in a microcentrifuge. Derivatization of thiol groups was performed using 10 µL of mBrB (Invitrogen, Oregon, USA), 25 mM, 450 µL of HEPES buffer (pH 8.2) with DTPA 6.3 mM and 250 µL of extract incubating in darkness for 30 min. To stop the reaction 300 µL of 1 M of MSA were added, samples were stored at 4 °C. The analysis of GSH and PCs were performed by HPLC coupled to FLD (Agilent, 1100 series); 20 µL of sample was injected and separated with a C18 column (5 µm, 4.6 × 150 mm) at 25 °C, using a binary mobile phase composed by 0.1% of TFA (aq) (A) and acetonitrile (B) in a linear gradient (0–20% of B in 10 min, 20–35% of B in 10 min and 35–100% of B in 10 min), using a flow rate of 1 mL min^−1^, FLD was setting in 380 nm of excitation wavelength and 470 nm of emission wavelength. Pure GSH (Sigma-Aldrich, St. Louis, MO, USA) and phytochelatins as used as standards, with polymerization degrees of 2–6 (AnaSpec Inc, San Jose, CA, USA) prepared in equal form that samples.

### Statistical analysis

All measures were analyzed using one-way ANOVA using Tukey’s post-test and statistical significance of *P* < 0.05.

## Results

### Antioxidant responses

**Fig. 1:**
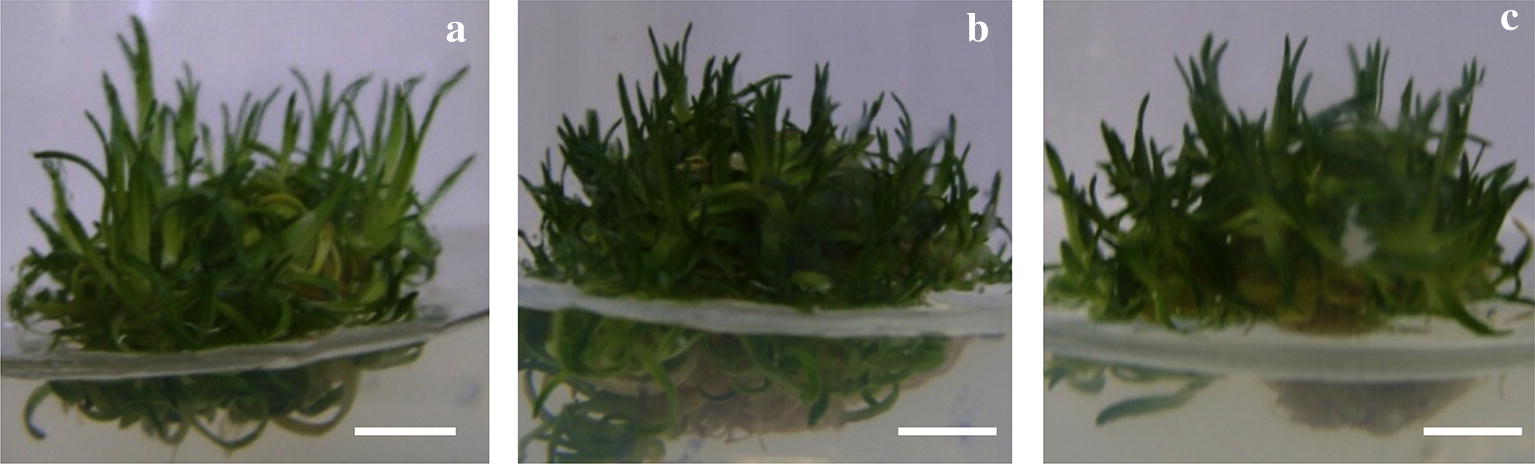
Pictures of *C. quitensis* at 15 days post-treatment. **a** Control condition, **b** subjected to 150 µM of copper and **c** subjected to 300 µM of copper. Bar represent 1 cm

**Fig. 2:**
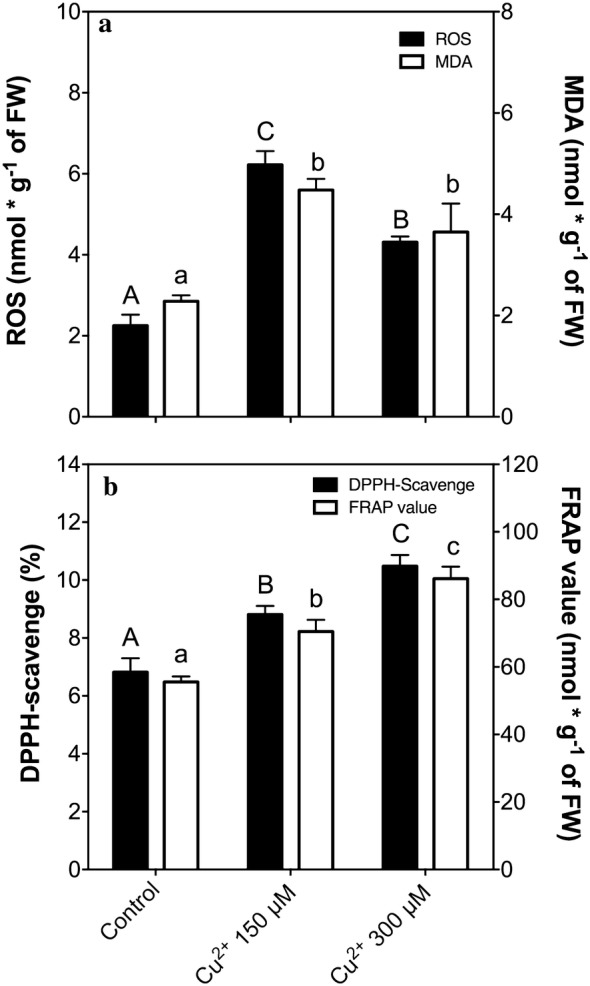
Oxidative stress parameters and non-enzymatic antioxidant activity. **a** ROS content (black bars) and MDA content (white bars) in *C. quitensis* subjected to copper and aluminum. **b** Non-enzymatic antioxidant activity as a DPPH-scavenging (black bars) and FRAP-value (white bars). Bars represent mean of three independent measurements. Significant differences were determined using ANOVA (P < 0.05)

The effect of supplementation with copper (150 and 300 µM) on oxidative parameters of in vitro cultures of *C. quitensis* is shown in Figs. 1 and 2. The total ROS accumulation shows significant increases in the treated plants, (Fig. 2a, black bars). Subsequently, the membrane peroxidation, measured as TBARS in MDA equivalents showing a similar pattern of accumulation in treated plants (Fig. 2a, white bars). The non-enzymatic antioxidant system measured as free radical-scavenge (DPPH assay, Fig. 2b, black bars) and as reducing power (FRAP assay, Fig. 2b, white bars), as complementary assays, shows an induction of non-enzymatic antioxidant machinery, as a concentration-dependent in copper (Fig. 2b), concluding that *C. quitensis* responds to treatments.

**Fig. 3:**
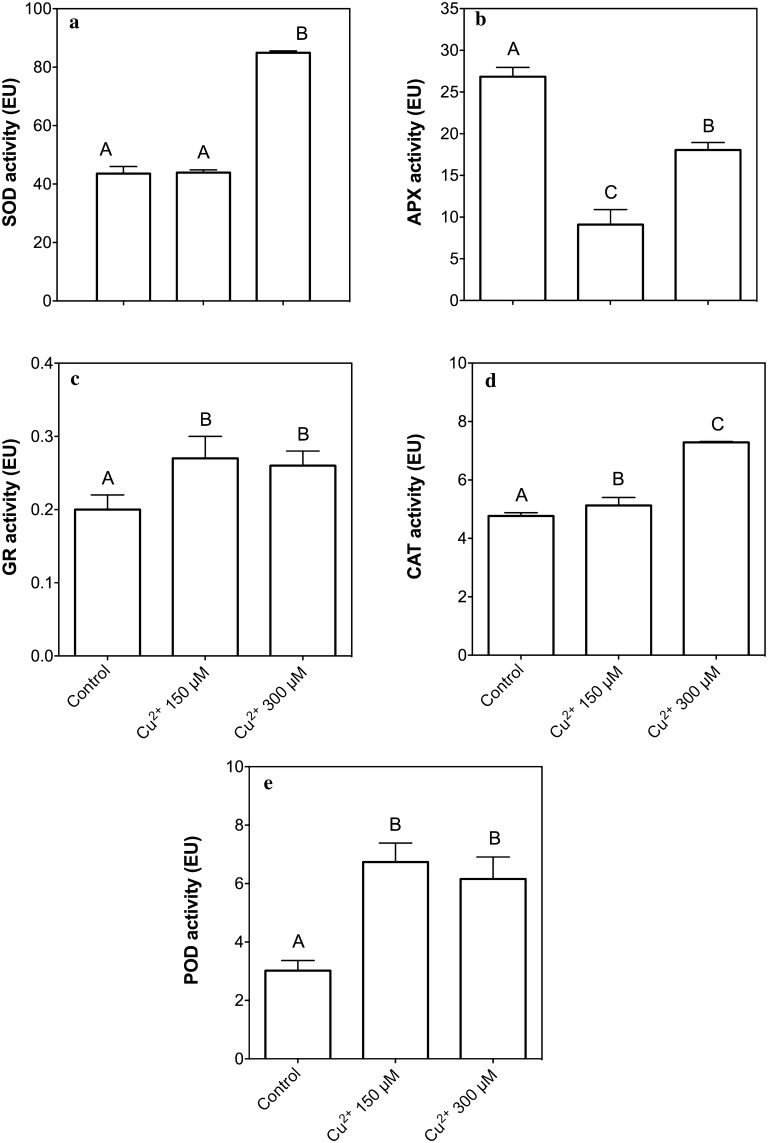
Activity of selected antioxidant enzymes. **a** SOD activity; **b** APX activity; **c** GR activity; **d** CAT activity and **e** POD activity. Bars represent mean of three independent measurements. Significant differences were determined using ANOVA (P < 0.05) with Tukey’s post-test, letters show significant differences

On the other hand, we analyze the enzymatic antioxidant system, an arrangement composed by five enzymes (SOD, APX, GR, CAT and POD; Fig. 3). The results showing that SOD (Fig. 3a), CAT (Fig. 3d) and POD (Fig. 3e) increase its activity in the copper treatments, however, APX (Fig. 3b) shows an inhibition in its activity in treated plants and GR (Fig. 3c) a measurable activity, with significant differences, but lowest than the other enzymes (from one to two magnitude orders), concluding an induction of antioxidant enzymes, but apparently does not involves the Asc-GSH cycle.

### Effect of copper in photosynthetic pigments, phenylpropanoids and sugars

**Fig. 4:**
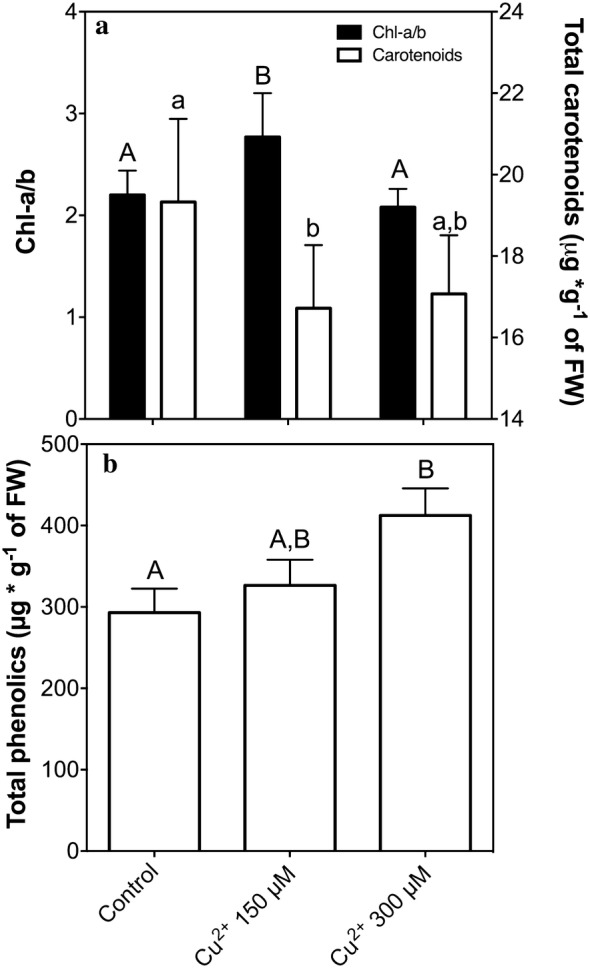
Photosynthetic pigments and content of phenolic compounds. **a** Photosynthetic pigments, *Chl*-a/b rate (black bars) and total carotenoids (white bars). **b** Total phenolics content according Folin-Ciocalteu’s. Bars represent mean of three independent measurements. Significant differences were determined using ANOVA (P < 0.05). In **b** with Tukey’s post-test, letters show significant differences

**Fig. 5:**
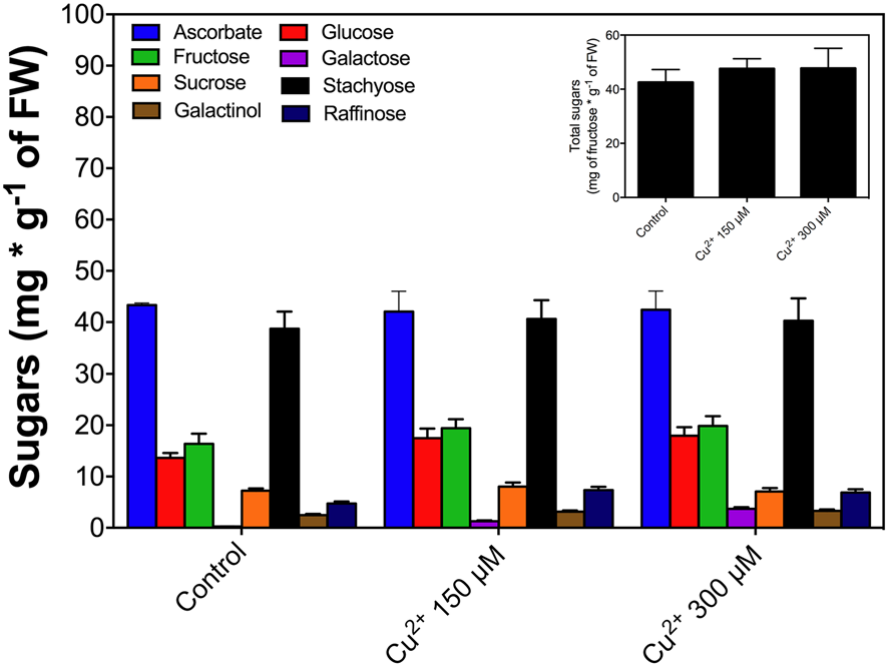
Carbohydrate content. Insert shows the total reducing content according reactivity with sulfuric anthrone. Grouped bars graph show different sugars detected by HPLC (symbols are showing in the graph). Bars represent mean of three independent measurements. Significant differences were determined using ANOVA (P < 0.05). In the insert with Tukey’s post-test, letters show significant differences

To evaluate the effect of copper in physiology of *C. quitensis* we measure photosynthetic pigments. The results showing that Chl-*a/b* ratio do not have significant differences in the treatments in comparison with control condition (Fig. 4a, black bars). Total phenolic compounds are increased in copper treatment in a concentration-dependent pattern (Fig. 4b), similar to non-enzymatic antioxidant activity, suggesting a role of soluble phenolics as antioxidants. Carbohydrates content measured as a total reducing sugars (anthrone method) does not showed significant differences between copper treated and control plants (Fig. 5 insert), in order to explain the real role of these sugars we analyze the profile of soluble sugars using HPLC. Glucose, galactose, raffinose and galactinol have significant differences with control conditions and are accumulated in a concentration-dependent pattern in copper treatment (Fig. 5). Other sugars such as fructose, sucrose and stachyose do not present significant differences with the control condition in copper treated plants (Fig. 5).

### GSH and PCs accumulation

**Fig. 6:**
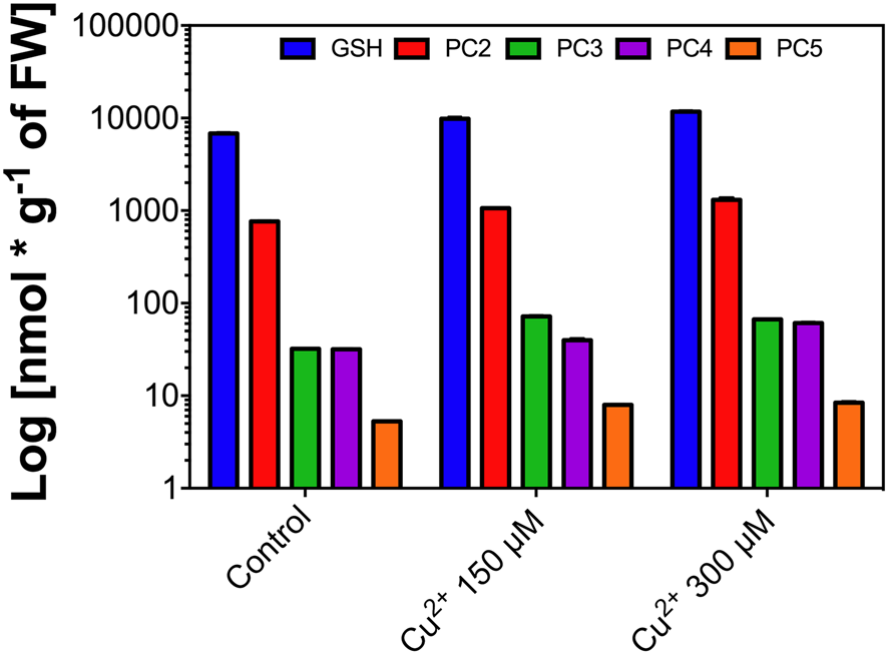
GSH and PCs content. Grouped bars showing different polymerization degrees of GSH detected by HPLC. Bars represent mean of three independent measurements. Significant differences were determined using ANOVA (P < 0.05). The graph is presented in a logarithmic scale

To demonstrate that the survivor capacity of *C. quitensis* against copper is governed by the described mechanisms for metal-tolerant species, we analyze the accumulation of GSH and PCs. The results showing that GSH, and PC_2_-PC_5_ are accumulated with significant differences with the control, in copper treated plants (Fig. 6).

## Discussion

### Antioxidant responses

Antioxidant enzymes reveals that both treatments induce the activity of SOD, CAT and POD, but APX activity decrease in the treatments while GR does not have significant differences, these results suggests that the Asc-GSH cycle does not important players in the detoxification of ROS in *C. quitensis* subjected to copper [35]. Some authors demonstrate that the presence of toxic substances such as cadmium inhibits APX activity, inactivating Asc-GSH cycle, this is a viable hypothesis could explain that the GR does not change, because APX is upstream in the detoxifying cycle [36, 37], for this reason all the antioxidant charge is conduced to alternative targets, such as non-enzymatic antioxidants. In this fact, the results showed increase in the free radical-scavenge and total reducing power, and are correlated with total phenolic content more than ascorbate and/or GSH, suggesting a role of phenolics as a free radical-scavengers acting in conjunct with SOD, CAT and POD to buffering the increased levels of ROS, has been discussed in literature the relevant role of phenolics such as flavonoids that acts interacting directly with ROS such as H_2_O_2_, for example [38, 39].

ROS causes different changes at cellular level, the most described in the literature is the interaction with unsaturated bonds in membrane lipids generating toxic aldehydes such as MDA [13], in the analysis of response of *C. quitensis* we demonstrate a direct correlation between ROS content and MDA content, but not sufficient to generates a lethal outcome, in the case of intracellular ROS in presence of metallic ions, has been described the generation of superoxide, and leads an increase in SOD activity [40], our results suggest that the excess of copper generates an increase of superoxide [41], increase the SOD activity generating H_2_O_2_ as a product, the H_2_O_2_ would be detoxified by CAT, POD and soluble phenolics [42–45].

Several authors described the role of H_2_O_2_ as a second messenger that controls different responses at cellular levels in different organisms, including plants [46]. The triggered signaling in copper stress in several species was characterized that controls both preventive and repairing systems, including antioxidant enzymes [47], GSH metabolism [48] and secondary metabolism [49], tonoplast transporters [50] and others. In this work, we characterize only at biochemical/physiological level, opening the possibility to characterize the response at molecular level, using next generation RNAseq, for example, to obtain information not only for the characterization of *C. quitensis* response, but also to obtain new blanks for molecular improvement of important sensible species (crop species, for example) and/or for phytoremediation of contaminated soils.

### Physiological parameters

Chl-*a/b* ratio does not show significant differences between control and treatments, this result suggest that the general photosynthesis was maintained in the presence of copper, this result supports the fact that *C. quitensis* is a multi-tolerant plant, because the physiological processes were not affected [51].

Phenolics as mentioned above, apparently acts as soluble scavengers of ROS, several authors define role of these molecules as antioxidants, such as flavonoids [52], phenolic acids, stilbenes, phenylpropanoids are good antioxidants and prevent oxidative damage *in planta* and in vitro [53–55].

### Sugar accumulation

Carbohydrates accumulation measured as a total reducing sugars does not shows significant differences in copper treatment compared with the control, for this reason we analyze by HPLC the profile of sugars, these results revealing significant differences in the content of raffinose and galactose. Raffinose contributes to homeostasis maintenance [56], in contrast, phenolic compounds showed an increase concentration dependent to copper dose, that acts as antioxidant molecules [54] and/or chelating molecules [57]. Galactose has been induced in a concentration dependent pattern, but galactose levels are lowest than other sugars, probably the increment in galactose and galactinol is a reflex of increment in biosynthesis of raffinose, a carbohydrate that acts as membrane stabilizing molecules [58]. We discarded the possible role of galactose to maybe act as precursor of ascorbate because the enzyme analysis reveals that the Asc-GSH cycle did not participate actively in the tolerance to copper and aluminum and ascorbate did not show changes along the treatments.

The crescent accumulation of galactose in both concentration, in form concentration-dependent is a possible result of galactolipid oxidation by ROS in chloroplast [59], the unbalance in redox status performed by the presence of abiotic elicitors in cellular environment led the membrane lipoperoxidation, in the case of chloroplast lipoperoxide derivatives, the liberation of toxic amounts of galactose has been reported previously in other species [60], the accumulation of raffinose inside the cells is a result of enhanced accumulation of galactose [59, 60] and the raffinose accumulation is a mechanism to remove the toxic amounts of galactose. In plants, raffinose is a key carbohydrate in the stress tolerance mechanisms [61], the accumulation of raffinose and galactinol is related to osmoprotection process in plants [62], moreover, raffinose plays other roles in plant tolerance to abiotic stress buffering ROS (act as soluble antioxidant), protecting for example, the radical hydroxylation of salicylate, and with other sugars, such as alditols, also acts as effective antioxidant compounds [62].

### GSH and PCs accumulation

The presence of copper induces the accumulation of GSH and PC_2_ to PC_5_. PC_6_ to PC_11_ has not been detected. GSH acts in three targets, first as soluble antioxidant compound that detoxifying directly the ROS accumulated inside the cell, second as an antioxidant cofactor of Asc-GSH cycle [63], however, in *C. quitensis* subjected to copper toxic levels this mechanism of detoxification does not participate in the tolerance, third, GSH chelate directly the toxic divalent cations, these complexes are translocated into the vacuole [64]. GSH is the precursor of PCs, they are polymeric forms of GSH and acts as strong chelating agents [59, 64, 65]. In the treatments with copper all detected phytochelatins are accumulated, PC_2_ and PC_4_ are concentration-dependent accumulated; meanwhile PC_3_ and PC_5_ are accumulated in concentration-independent form in response to excess of toxic cations, demonstrating the potential of *C. quitensis* to faces the presence of toxic elements like tolerant species [66] and demonstrate that *C. quitensis* is a good model of a multi-tolerant plant.

Antarctic soils showed a mean concentration of cupric ion of 2.0 ± 0.27 mmol kg^−1^ (124 ± 17 ppm) in soils of King George Island [3], normally, uncontaminated soils have a concentration of 1.5 mmol kg^−1^ or less of cupric ion [67] and contaminated sites (highly intervened) such as exploited copper mines have high concentrations nearby 5–8 mmol kg^−1^ of cupric ion [67, 68], this analysis reveals that Antarctic soil (in King George Island) have moderated contamination, capable to generate a defensive response in *C. quitensis*. Our results suggest that the redundant capacity of antioxidant system to face oxidative stress (product of different abiotic extreme conditions) and the capacity to accumulate phytochelatins are important in the survival of *C. quitensis* in the field.

Another fact, but not less important, is that Caryophyllaceae family plants are classified as hyperaccumulators and/or tolerant species in terms of metallic ion accumulation. In *Silene vulgaris* (syn. *S. cucubalus*) the tolerant variety survive to 250 µM of cadmium, accumulating 12–13 µmol g^−1^ of PC_2_ [69, 70], similar levels than *C. quitensis* exposed to 300 µM of copper. Another study, demonstrates the growth of *S. dioica* in copper mines, highly contaminated soils (> 8 mmol kg^−1^ of cupric ion in soil) [71]. Contrasting the literature and our results about *C. quitensis* (exposed to moderated concentrations of cupric ion in field), we hypothesize that the capacity of *C. quitensis* to tolerate in vitro high concentrations of cupric ions, probably was acquired for a primitive ancestor of Caryophyllaceae family that inherited their copper tolerance capacity to modern members of their family such as *Silene* sp. and *C. quitensis*.

## Conclusions

*C. quitensis* exhibits a natural capacity to tolerate high levels of cupric ion in vitro. The mechanisms behind their capacity involves antioxidant machinery and GSH derivative compounds (phytochelatins). Our results suggest the projection of *C. quitensis* as a multi-tolerant specie to several abiotic conditions and bring out this specie as a model to investigate their capacity at the molecular level.

## Abbreviations

OH·: hydroxyl radical
^1^O_2_: singlet oxygen
APX: ascorbate peroxidase
Asc: ascorbate
BSA: bovine seroalbumine
CAT: catalase
Chl: chlorophyll
Cys: cysteine
DCF: oxidized dichlorofluorescein
DCHF-DA: dichlorodihydrofluorescein diaceatete
DNA: deoxyribonucleic acid
DPPH.: 1,1-diphenyl-2-picrilhydrazil radical
DPPH_2_: 1,1-diphenyl-2-picrilhydrazine
DTPA: diethylenetriamine pentaacetic acid
EDTA: ethylenediamine tetraacetate
FLD: fluorescence detector
FRAP: ferric reducing/Antioxidant power assay
Glu: glutamate
Gly: glycine
GPX: glutathione peroxidase
GR: glutathione reductase
GSH: reduced glutathione
GSSG: oxidized glutathione dimer
H_2_O_2_: hydrogen peroxide
HPLC: high performance liquid chromatography
MBrB: monobromobimane
MDA: malondialdehyde
MSA: methanesulphonic acid
MTs: metallothioneins
NA: nicotianamine
NADPH: nicotinamide dinucleotide phosphate reduced
NBT: nitroblue tetrazolium
$\text{O}_{2}^{ \cdot - }$: superoxide anion radical
PAR: photosynthetically active radiation
PC_n_: phytochelatin n (n = polymerization degree)
PCs: phytochelatins
POD: type III peroxidase (syn.: guaiacol peroxidase)
PSI: photosystem I
PSII: photosystem II
RID: refraction index detector
ROS: reactive oxygen species
SOD: superoxide dismutase
TBA: thiobarbituric acid
TBARS: thiobarbituric acid reactive substances
TCA: trichloroacetic acid
TFA: trifluoroacetic acid
TPTZ: 2,4,6-tris(2-pyridil)-*s*-triazine
UV-B: ultraviolet B radiation
YSL: yellow stripe1-like
